# Equivalent efficacy of indoor daylight and lamp‐based 5‐aminolevulinic acid photodynamic therapy for treatment of actinic keratosis

**DOI:** 10.1002/ski2.226

**Published:** 2023-03-31

**Authors:** Alberto J. Ruiz, Ethan P. M. LaRochelle, Marie‐Christine P. Fahrner, Jennifer A. Emond, Kimberley S. Samkoe, Brian W. Pogue, M. Shane Chapman

**Affiliations:** ^1^ Thayer School of Engineering at Dartmouth Hanover New Hampshire USA; ^2^ Department of Dermatology Geisel School of Medicine at Dartmouth Hanover New Hampshire USA; ^3^ Dartmouth Geisel School of Medicine Hanover New Hampshire USA

## Abstract

**Background:**

Photodynamic therapy (PDT) is widely used as a treatment for actinic keratoses (AK), with new sunlight‐based regimens proposed as alternatives to lamp‐based treatments. Prescribing indoor daylight activation could help address the seasonal temperature, clinical supervision, and access variability associated with outdoor treatments.

**Objective:**

To compare the AK lesion clearance efficacy of indoor daylight PDT treatment (30 min of 5‐aminolevulinic acid (ALA) pre‐incubation, followed by 2 h of indoor sunlight) versus a lamp‐based PDT treatment (30 min of ALA preincubation, followed by 10 min of red light).

**Methods:**

A prospective clinical trial was conducted with 41 patients. Topical 10% ALA was applied to the entire treatment site (face, forehead, scalp). Patients were assigned to either the lamp‐based or indoor daylight treatment. Actinic keratosis lesion counts were determined by clinical examination and recorded for pre‐treatment, 1‐month, and 6‐month follow‐up visits.

**Results:**

There was no statistical difference in the efficacy of AK lesion clearance between the red‐lamp (1‐month clearance = 57 ± 17%, 6‐month clearance = 57 ± 20%) and indoor daylight treatment (1‐month clearance = 61 ± 19%, 6‐month clearance = 67 ± 20%). A 95% confidence interval of the difference of the means was measured between −4.4% and 13.4% for 1‐month, and −2.2% and +23.6% for 6‐month timepoints when comparing the indoor daylight to the red‐lamp treatment, with a priori interval of equivalence of ±20%.

**Limitations:**

Ensuring an equivalent dose between the indoor and lamp treatment cohorts limited randomisation since it required performing indoor daylight treatments only during sunny days.

**Conclusion:**

Indoor‐daylight PDT provided equivalent AK treatment efficacy to a lamp‐based regimen while overcoming temperature limitations and UV‐block sunscreen issues associated with outdoor sunlight treatments in the winter.

**Clinical trial registration:**

Clinicaltrials.gov listing: NCT03805737.

## Graphical abstract

Indoor daylight photodynamic therapy (PDT) for actinic keratoses (AKs) could overcome the treatment limitations associated with lamp‐based PDT and outdoor daylight treatments. This study shows AK clearance equivalency between indoor daylight and red‐lamp PDT treatments, such that its adoption could enable accessible, well‐tolerated, treatments throughout all seasons.

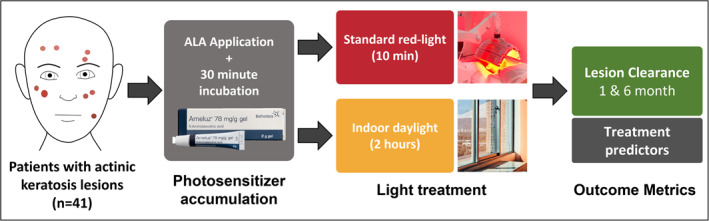

1

**What is already known about this topic?**

- Photodynamic therapy (PDT) is widely used as a treatment for actinic keratoses (AK), with new sunlight‐based regimens proposed as alternatives to lamp‐based treatments.

**What does this study add?**

- This study is the first to examine efficacy of daylight PDT treatment through a window (indoor daylight), combining benefits of traditional inpatient lamp‐based treatments and outdoor daylight‐based regimens. Adoption of this treatment modality within the standard‐of‐care could expand PDT treatment to at‐home and outpatient clinic settings, increasing the accessibility and affordability of PDT in dermatology.

**What is the translational message?**

- This study shows AK clearance equivalency between indoor daylight and red‐lamp PDT treatments, such that its adoption could enable accessible, well‐tolerated, treatments throughout all seasons.

## 2 INTRODUCTION

Photodynamic therapy (PDT) is one of the most widely used treatments for actinic keratoses (AKs) worldwide,^1^ and especially useful for multiple lesion treatment.^2^ While the original clinical approvals for treatment with PDT have been with lamp light activation, there has been adoption of using outdoor daylight to activate the treatment for more than a decade.^3^, ^4^ During this time, many countries have gone through trials and developed their own consensus statements for the use of daylight PDT.^5^, ^6^, ^7^ Nevertheless, the issues remain around the control of the patient, reliability of the sunlight, and temperature during the daylight exposure, especially in the winter and summer months.^8^, ^9^ More recent studies have demonstrated that it should be possible to achieve equivalent light dose with indoor‐daylight PDT,^10^, ^11^ which provides a treatment where the environment can be better monitored. Furthermore, adoption of indoor daylight treatments could transition into at‐home treatment of patients.

Daylight PDT has been beneficial for several reasons, including pain management^12^ and patient comfort.^13^ However, its clinical acceptance has been limited due to concerns around weather and seasonal fluctuations, each of which makes the available sunlight exposure and temperature variable.^8^, ^9^ This is particularly problematic in higher latitudes, where a dominant fraction of AK treatments are provided.^14^ Additionally, within certain countries such as the USA, the ability to gain reimbursement for the treatment can be tied into ensuring that the treatment is done inside a clinic, where there is control over patient participation and the delivery of therapy. For these reasons, there has been an increased interest in performing daylight PDT indoors where the variables of temperature and patient compliance can be minimised.^15^, ^16^ It is worth noting that in literature the term “indoor‐daylight” is often incorrectly used to describe the practice of low irradiance white‐lamp treatments, here this term is used as the delivery of daylight through a window with a patient located within an indoor space. Two major technical adoption roadblocks of indoor‐daylight PDT involve sunlight surveying planning and accounting for changes in patient irradiation with varying weather conditions. The assessment of effective treatment dose equivalency between PDT light sources and accounting for weather‐based variation is necessary for widespread implementation of daylight‐based treatments.

In this study, the concept of daylight PDT was further expanded by testing treatment in a controlled prospective clinical trial that delivered indoor daylight PDT, using Ameluz® (Biofrontera), during the winter months in Northeastern United States. The study used daylight treatment inside the dermatology clinic, with an exposure that was surveyed and confirmed to have sufficient light fluence for effective activation.^10^ The control group was a clinically used, well‐tolerated delivery, of red‐lamp PDT with standard light fluence. The trial was carried out through the winter months to test the efficacy of dermatology clinics with a northern latitude, with the hypothesis that indoor daylight PDT would demonstrate equivalent AK lesion clearance compared to lamp‐based PDT.

## 3 METHODS

### 3.1 Study design

**FIGURE 1:**
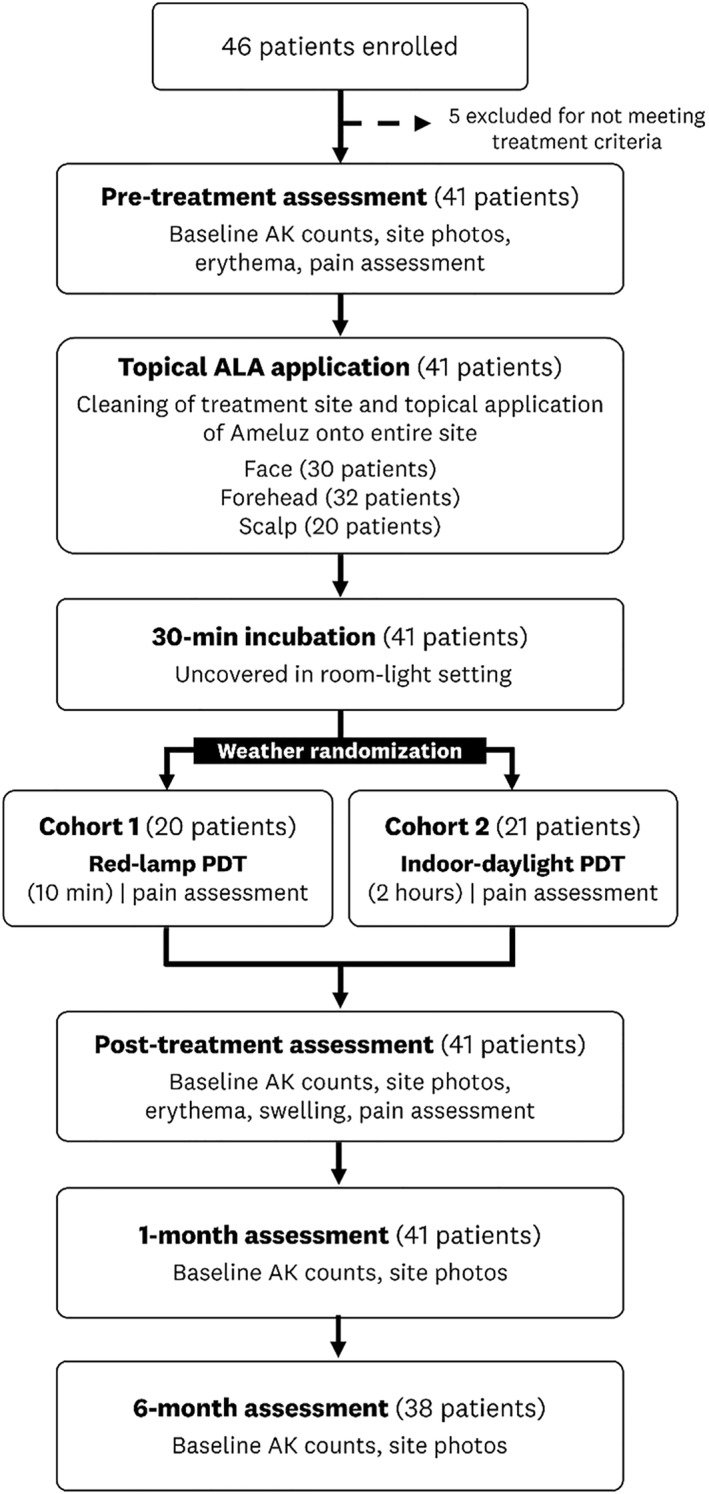
Study design with patient enrolment numbers at each stage.

This prospective clinical trial was conducted at a single dermatology clinic (Dartmouth‐Hitchcock Medical Centre, Department of Dermatology, [Latitude, Longitude] = [43.65, −72.24]). Participants were recruited from the standard patient population appearing with AKs at the Dartmouth‐Hitchcock Department of Dermatology Clinic and gave informed consent before enrolment. Figure 1 shows a visual summary of the study design alongside patient enrolment numbers at each stage. The study was approved by the Dartmouth‐Hitchcock Health Institutional Review Board and registered with ClinicalTrials.gov (NCT03805737).

### 3.2 Study population

Inclusion criteria were patients aged ≥18 years appearing at the Dartmouth Dermatology Clinic for PDT treatment of AKs. Candidates with conditions that were not suitable for clinical PDT, including taking photosensitising medication and patients with a history of melanoma in the treatment site, were excluded from the study. Enroled patients had at least 5 AK lesions (grade I and II, Olsen classification) on the treatment sites (face, forehead, scalp).

### 3.3 Randomisation

Patients were randomly assigned either red‐lamp or indoor daylight treatment to keep number parity between treatment arms. For this study, randomisation was limited due to the weather dependence of the indoor daylight PDT treatment. To provide equivalence in the delivered dose to the red‐lamp treatment, patients were enroled for the indoor daylight PDT cohort only on days with “sunny” to “mostly‐sunny” conditions to ensure sufficient radiant exposure; on cloudy days, patients were always assigned to the red‐light treatment. Patients were instructed to cover the treatment site and avoid sunlight for 48 h after irradiation, which minimised the confounding effects of enrolling daylight PDT patients exclusively on sunny days. The red‐light treatment was used as the control in this study.

### 3.4 Treatment

Cleaning of the treatment area (face, forehead, and scalp) using a 70% isopropyl solution was followed by topical application of a 10% ALA gel (Ameluz®, Biofrontera). Post ALA application, patients were incubated under room‐light conditions for 30 min, with the area of treatment uncovered. Following the ALA incubation, light irradiation was performed on the treatment areas. The lamp‐based PDT cohort was irradiated for 10 min using red‐light (BF‐RhodoLED, Omnilux, 635 nm, measured irradiance 57 mW/cm^2^, 0.96 mW/cm^2^ PpIX‐effective irradiance). The indoor‐daylight PDT cohort sat in front of an east‐facing window where they received direct sunlight for 2 h (measured irradiance 15 mW/cm^2^, 1.1 mW/cm^2^ PpIX‐effective irradiance). The treatment window was chosen following sunlight surveying,^10^ which identified a 4‐h time period where patients could be treated with direct sunlight through the window. The patient placement was checked every 30 min and adjusted when necessary to account for the sun's movement throughout the treatment. Following the light irradiation, sunblock was applied to the treated sites, and the patients were instructed to avoid sunlight for the next 48 h.

### 3.5 Radiant exposure and effective dose

Irradiance spectra for the RhodoLED lamp and indoor daylight were measured using a spectroradiometer (Sekonic Spectromaster C800). The PpIX‐effective irradiance spectra was is calculated by multiplying the irradiance spectra by the normalised PpIX absorption spectra.^17^ Effective fluence was calculated using light penetration curves from a 7‐layer skin model.^11^, ^18^ The resulting radiant exposure and effective dose for red‐light (10 min) and indoor daylight (2 h) treatments are calculated by integrating over the irradiance and fluence spectra and are summarised in Table 1 below. The full PpIX‐effective fluence and dose versus skin depth for the red‐lamp and indoor‐daylight treatments are plotted in Figure S1. The effective PpIX dose calculations show that the indoor‐daylight treatment provides a higher dose (2X) for depths below <250 μm, which corresponds to the location of the upper blood dermis in the 7‐layer model. The delivered PpIX‐effective dose is equivalent within 20% in the 350–1000 μm range, with an equal dose delivered at 700 μm between the two treatment regimens.

**TABLE 1:** Radiant exposure and effective dose for the RhodoLED lamp (10 min) and indoor daylight (2 h) treatments.

	Radiant exposure (kJ/m^2^)	Effective radiant exposure (kJ/m^2^)	Effective dose (kJ/m^2^) @ depth (μm)							
			200	500	750	1000	1250	1500	1750	2000
RhodoLED (10 min)	342.1	5.8	6.3	6.5	5.8	4.9	4.0	3.3	2.5	2.3
Indoor daylight (2 h)	1064.1	76.7	14.7	7.8	5.6	3.9	2.8	2.0	1.4	1.1

The reported dose from the indoor daylight is calculated from the irradiance spectra from a representative “sunny” condition during treatment. The irradiance of treatment was tracked using local weather irradiance reports, showing an average outdoor solar radiance of 36 ± 9 mW/cm^2^ for the daylight‐treated patients. Compared to these reported irradiances, the measured patient irradiance was ∼45% which is attributed to the angular dependance of solar irradiance and a measured window transmission of ∼62% for the solar spectrum.

**FIGURE 2:**
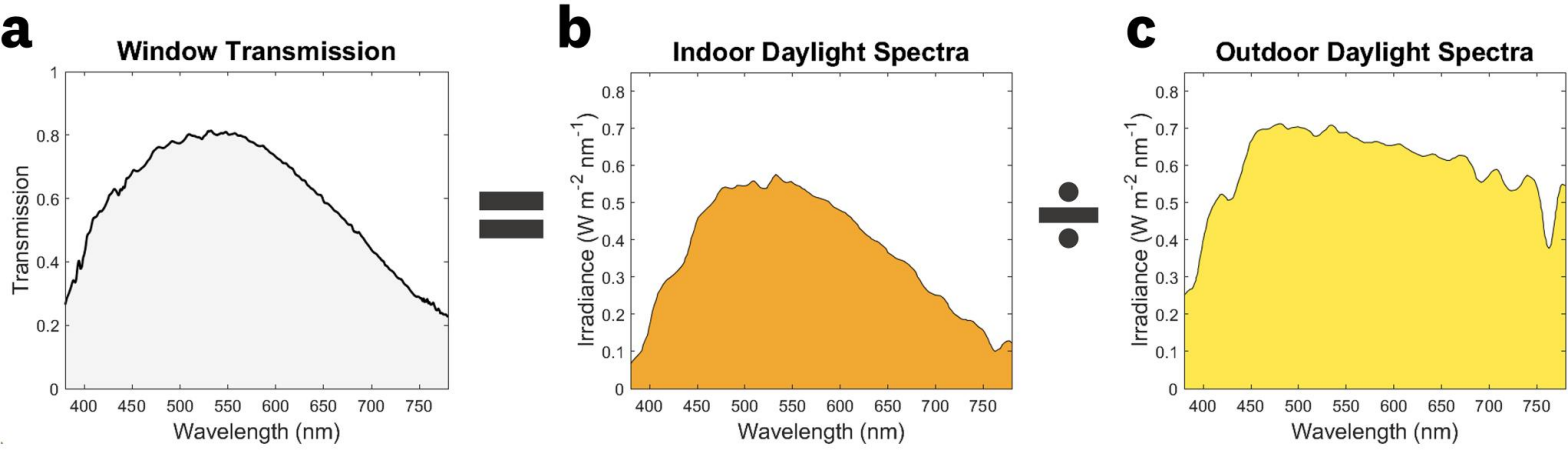
Window transmission spectra. The (a) window transmission spectrum is calculated from simultaneously acquired (b) indoor daylight, and (c) outdoor daylight spectra.

Figure 2 shows the calculated window transmission spectrum, derived from the measured outdoor daylight and indoor daylight spectra. The calculated window transmission spectrum shows attenuation of UV and NIR wavelengths, which are a result of glass substrate absorption (UV wavelengths) and infrared‐reflective coatings. This reduction in indoor irradiance comes with the benefit of blocking UVC (100–280 nm) and UVB (280–315 nm) radiation while minimising UVA (315–400 nm) radiation for indoor treatments compared to outdoor‐based treatments.

### 3.6 Outcome measures

The primary outcome was therapeutic efficacy (AK lesion clearance), defined as the reduction in the number of AK lesions between the baseline (day of treatment) and the follow‐up visit (1‐month and 6‐month post‐treatment). The AK lesions were photographed, mapped, and counted by physicians for the treatment region (face, forehead, scalp) for both the baseline, 1‐month, and 6‐month follow‐up.

Pain assessment and erythema resulting from the PDT treatment were measured as secondary outcomes. For pain assessment, the patients were asked to report their pain on a 0‐to‐10 visual analog scale (Supplemental Material Document in Supporting Information S1) throughout the treatment (at baseline, after incubation, and throughout the light irradiation). At baseline and post‐treatment, a physician graded erythema on a 0–4 scale (Supplemental Material Documentin Supporting Information S1). The physicians were blinded on the patient's treatment cohort.

### 3.7 Statistical analysis

The AK clearance rate was calculated from the physicians assessments of the number of AK lesions (type I and II) in the treatment site during the pre‐treatment, 1‐month, and 6‐month timepoints for each patient. The two‐sample *t‐*test was used to determine difference in mean clearance rate between the lamp and indoor daylight treatment cohorts. The two‐sample equivalence test was used to determine if the 95% confidence interval for the difference in the test and control group means was within the prespecified zone of equivalence, defined as ±20%.

Associations between patient metrics (age, number of lesions) and the secondary outcomes (pain, erythema) to the therapeutic efficacy of the treatment were visually assessed with scatterplots; Pearson's correlation coefficients were computed to assess the strength of linear associations. Those analyses were completed for the sample overall and stratified by treatment assignment. Statistical significance was considered at a *p* value of less than 0.05. All analyses were performed with Minitab 20.1 (Minitab LLC, PA, USA).

### 3.8 Sample size justifications

The study was powered to evaluate the equivalency of AK lesion clearance rate between indoor daylight and red‐lamp PDT, with the effect size and variability in response estimated from previous red‐lamp PDT studies.^19^, ^20^ A sample size of 20 patients per treatment arm was chosen to show equivalency within a ±20% range, assuming a standard deviation of 20%, resulting in a power >80% with an alpha value of 0.05.

## 4 RESULTS

Forty‐six patients with actinic keratosis lesions were enroled, with five excluded from the study (Figure 1). Of the remaining 41 patients, 29 were men (mean age: 72 years, range: 55–79 years) and 12 women (mean age: 70, range: 60–77 years). All patients were White with an average number of 16 lesions (standard deviation: 8 lesions, range: 5–50 lesions) in the treatment area. The lesions were found in the face for 30 patients, the forehead for 32 patients, and the scalp for 18 patients.

Red‐lamp PDT was performed on 20 patients and indoor‐daylight PDT treatment on 21 patients. The mean (SD) outdoor temperature during treatment times was 0.4 °C (9.2 °C) with indoor temperatures of 23 °C (2 °C). Table 2 summarises the baseline patient characteristics and clinical outcomes of the study. Additional plots visualising individual patient values across all measurements are provided in Figure S3. No statistical difference was found between the study populations of the two treatment cohorts (age, skin type, and lesion numbers).

**TABLE 2:** Patient characteristics and clinical outcomes.

*N* = 41 Patients	Study	Red‐lamp (*N* = 20)^a^	Indoor‐daylight (*N* = 21)	*p*‐value
Sex				
Male	29 (71%)	15	14	
Female	12 (29%)	5	7	
Age (years)	71 ± 6	72 ± 7	71 ± 6	0.67
Skin type (Fitzpatrick)	1.8 ± 0.4	1.8 ± 0.4	1.9 ± 0.4	0.43
Lesions (Day 0)	16 ± 8	17 ± 7	15 ± 10	0.56
Pain rating (max reported)		1.8 ± 1.8	1.5 ± 1.4	0.43
Change in erythema		0.6 ± 0.6	1.1 ± 0.6	0.005
Lesions (1 month)		8 ± 4	6 ± 5	0.43
Lesion clearance rate (1 month)		57 ± 17%	61 ± 19%	0.42
Lesions (6 months)^a^		7 ± 4	5 ± 6	0.30
Lesion clearance rate (6 months)^a^		57 ± 25%	68 ± 20%	0.17

*Note*: Values reported as mean ± SD.

^a^ The total number of patients included in the 6‐month timepoint is 38, losing 3 to follow‐up from the 1‐month timepoint.

The mean (SD) AK lesion clearance rate at the 1‐month follow‐up was 57% (17%) for red‐lamp PDT and 61% (19%) for the indoor daylight treatment. There was no statistical difference (*p* = 0.42, 2‐sample *t*‐test) in the efficacy of AK lesion clearance between the two treatment arms. The calculated 95% CI of the difference in AK lesion clearance means was between −4.4% and +13.4% when comparing indoor daylight treatment to the red‐lamp treatment at the 1‐month time point, falling within the defined ±20% zone of equivalence.

The mean (SD) AK lesion clearance rate at the 6‐month follow‐up was 57% (25%) for red‐lamp PDT and 68% (20%) for the indoor daylight treatment. There was no statistical difference (*p* = 0.17, 2‐sample *t*‐test) in the efficacy of AK lesion clearance between the two treatment arms. The calculated 95% CI of the difference in AK lesion clearance means was between −2.2% and +23.6% when comparing indoor daylight treatment to the red‐lamp treatment at the 6‐month endpoint, falling marginally outside of the defined ±20% zone of equivalence.

The max reported pain during treatment was not statistically different (*p* = 0.43) between the two treatment cohorts with average reported pain (visual‐analog‐scale, 0–10) of 1.8 and 1.5 for the red‐lamp and indoor daylight treatments, respectively.

There was a statistically significant difference in the reported change in erythema (*p* = 0.005, 2‐sample *t*‐test) between the treatment cohorts (0–4 scale) with mean (SD) changes of 0.6 (0.6) and 1.1 (0.6) for the red‐lamp and indoor daylight treatments, respectively. The resulting difference in change in erythema is most likely due to higher radiant exposure associated with the indoor daylight treatment (Table 1).

The corresponding individual value plots for the patient characteristics and clinical outcomes reported in Table 2 are shown in Figure S2. These plots show the heterogeneity in the primary (AK lesion clearance) and secondary (pain rating, erythema) outcomes for patients in both treatment cohorts.

The association between AK lesion clearance to patient characteristics and secondary outcomes are shown in Figure S3 for the entire study cohort. The plots include corresponding statistical significance (*p*‐values) and Pearson correlation coefficients. For both the 1‐month clearance [Figure S3a] and 6‐month clearance [Figure S3b] age, pre‐treatment lesion numbers, and pain rating showed no statistical significance as a predictor of AK clearance rate with no to minimal correlation (Pearson coefficient < |0.2|). Only erythema showed statistical significance within the 1‐month lesion clearance, which was primarily driven by an outlier patient that had the largest change in erythema along with a 100% lesion clearance rate (*r* = 0.32, *p* = 0.04).

The association between the 6‐month clearance rate and patient characteristics and secondary outcomes, stratified by treatment arm assignment, are presented in Figure S4. Among those in the red‐lamp treatment [Figure S4a], none of the correlations were statistically significant and effect sizes were small (*r* < |0.18|) except for the correlation between 6‐month clearance rate and age which was a medium effect size, although not statistically significant (*r* = 0.30; *p* = 0.25). For the indoor‐daylight cohort [Figure S4b], the reported pain rating had moderate correlation (Pearson coefficient >0.4) with borderline statistical significance (*p*‐value = 0.055). The age, number of baseline lesions, and erythema were not statistically significant predictors for the indoor‐daylight cohort.

Site‐specific (scalp, forehead, and face) AK clearance rates for the 1‐month and 6‐month timepoints were also calculated for each cohort [Table 3]. A schematic for the categorical location of face, forehead, and scalp lesions is provided in Figure S6 with site‐specific lesion numbers reported in Table S1. For the 1‐month time point, the scalp lesions had a lower mean clearance (>10%) than the forehead and face lesions for both the red‐lamp and indoor‐daylight cohorts. Comparing the 1‐month indoor daylight forehead and scalp clearance rates showed borderline statistical significance (*p* = 0.053, 2‐sample *t* test). The lower clearance rate for scalp lesions persists into the 6‐month timepoint. Compared to the 1‐month timepoint, the 6‐month site clearance rates for both treatment arms had a small (3%) to moderate (13%) increase. The site‐specific data shows that the forehead lesion clearance rate drives the differences observed in the overall lesion clearance observed between the two treatment cohorts at the 1‐month (4% difference) and 6‐moth (11% difference) time points (Table 2).

**TABLE 3:** Site‐specific lesion numbers and clearance rates for each treatment arm for 1 and 6 months timepoints.

Timepoint	Site	Red‐lamp treatment	Indoor‐daylight treatment
Pre‐treatment lesions (mean ± SD)	Face	6 ± 2	7 ± 3
	Forehead	6 ± 3	6 ± 4
	Scalp	10 ± 7	13 ± 5
1‐month post‐treatment clearance rate (mean ± SD)	Face	60 ± 15%	65 ± 22%
	Forehead	59 ± 23%	70 ± 20%
	Scalp	49 ± 33%	46 ± 32%
6‐month post‐treatment clearance rate (mean ± SD)	Face	69 ± 35%	72 ± 16%
	Forehead	65 ± 28%	73 ± 31%
	Scalp	60 ± 39%	59 ± 26%

## 5 DISCUSSION

Here, we report the results from the first clinical trial designed to compare the efficacy of indoor sunlight‐based PDT treatment to a lamp‐based treatments. The results measured a 95% CI for the difference of means for the 1‐month AK lesion clearance of −4.4%–13.4%, and a 6‐month clearance between of −2.2% and +23.6% when comparing the indoor‐daylight treatment to the red‐lamp regimen. Based on the a priori ±20% interval of equivalence, the results show noninferiority for the indoor treatment and suggests it may in fact be superior to the red‐lamp treatment based on the 6‐month AK clearance rates. This indicates that indoor daylight treatment through a window should be a suitable alternative to lamp‐based treatment of AK lesions. Indoor daylight PDT treatments can help address the cost limitations of lamp‐based PDT while overcoming the temperature limitations of outdoor sunlight treatments in both cold winter and hot summer days. Furthermore, the indoor daylight treatment through a window eliminates the need to monitor the UV exposure associated with outdoor daylight PDT given the inherent filtering of UV‐A and UV‐B rays.^18^, ^21^

The 30‐min ALA incubation time with a 10‐min red‐light treatment was chosen for our comparison control group because it is widely practiced in Dermatology centres and is a well‐tolerated regimen, including within Dartmouth‐Hitchcock Department of Dermatology. It is important to note that this is in contrast with the suggested Ameluz® treatment protocol of 3‐h incubation with occlusion. However, the 30–60 min incubation, as well as simultaneous illumination, has been adopted in other clinical studies and has become common in clinical practice because it provides shorter visit times and lower reported pain of treatment.^1^, ^19^, ^22^, ^23^, ^24^, ^25^ The mean clearance rate and standard deviations reported in this study match the results of similar lamp‐based protocols.^19^, ^20^ The 2‐h treatment for the indoor daylight cohort was based on standard practice in outdoor daylight protocols. The measured irradiance spectra for the red‐lamp and indoor‐daylight, alongside skin fluence calculations, showed similar delivered dose for the patients in this study. It is worth noting that the indoor‐daylight cohort had a similar variance in AK lesion clearance rates as the red‐lamp treatment, despite the monitored outdoor irradiance indicating a potential ∼25% variation in the daylight dose provided to patients.

The results also indicate no statistical difference in the average patient assessment of pain between the two treatment cohorts. The only statistically significant difference was found in the change of erythema, where the indoor daylight cohort observed larger changes at the end of treatment. This difference is most likely due to the higher radiant exposure from the indoor daylight treatment. Follow‐up on erythema in subsequent days was not available in this trial study.

Correlation for assessment of lesion clearance predictors were performed for patient age, skin type, baseline lesion number, maximum pain rating, and change in erythema. The red‐lamp PDT cohort showed minimal to no correlation for all factors with no statistically significant regression. The indoor daylight cohort showed moderate correlation (*r* > |0.4|) and borderline statistical significance for the reported pain ratings (*p* = 0.055).

The site‐specific lesion clearance measurements showed lower clearance rates for the scalp lesions for both treatment cohorts. These lower clearance rates for scalp lesions match the results of other PDT studies.^19^ The higher average lesion clearance values for the daylight cohort were driven by higher rates of facial and forehead lesion clearance. For the daylight cohort, the forehead lesions had the highest clearance rates, which might result from the relatively even surface irradiation compared to the curvature of the face allowing for more homogeneous dose delivery.

The main limitation of this study was the weather dependence for the daylight treatments, which could provide confounding variables since the daylight cohort is assigned only on sunny‐mostly sunny days (such that patients could further receive irradiation outside of the clinic). To minimise this effect, patients were instructed to keep the treatment sites covered and away from sunlight for 48 h.

Multi‐centre studies for indoor daylight treatment would be a way to further establish the efficacy of indoor daylight PDT treatment. Such a multi‐centre effort might ideally be set up with careful characterisation of sunlight irradiation at each study site to ensure consistent treatment dose.^9^ Future studies could monitor the indoor‐daylight radiant exposure alongside the PpIX‐effective dose at varying depths to further understand the impact of the delivered dose on daylight PDT treatment outcomes. Additionally, a de‐escalation study for indoor daylight treatment, including 1 versus 2 h irradiation, could help minimise treatment times. Measurement of the PpIX accumulation during and post light irradiation could also help determine the heterogenous response among individual patients.^26^, ^27^ Adoption of at‐home PDT treatment using indoor daylight might also be possible as a future delivery method, requiring site assessments for dose determination and tele‐health monitoring of the treatment.

## 6 CONCLUSIONS

Indoor daylight PDT appears to be an acceptable method for delivering AK lesion treatment as a result of its equivalent efficacy to lamp‐based PDT and tolerable side effect profile. This approach helps overcome costs and seasonal limitations associated with lamp‐based and outdoor daylight PDT. Given the well‐known geographic overlap between patients with AK incidence and their location in northern hemisphere countries that experience substantial winter, this paradigm of indoor daylight PDT would be a practical way to approach future treatments. Furthermore, adoption of indoor daylight PDT could enable at‐home treatment of patients, particularly for those in rural areas. This study provides the initial steps in the implementation of indoor‐daylight treatments within clinics while providing estimates of AK lesion clearance rates, clearance variance, and daylight dose calculations to help guide future indoor‐daylight clinical studies.

## CONFLICT OF INTEREST STATEMENT

None declared.

## AUTHOR CONTRIBUTIONS

**Alberto J. Ruiz**: Conceptualization; Data curation; Formal analysis; Investigation; Methodology; Validation; Visualization; Writing original draft. **Ethan P. M. LaRochelle**: Data curation; Formal analysis; Validation. **Marie‐Christine P. Fahrner**: Data curation; Investigation; Project administration; Resources; Supervision. **Jennifer A. Emond**: Data curation; Formal analysis; Validation; Writing review & editing. **Kimberley S. Samkoe**: Formal analysis; Writing review & editing. **Brian W. Pogue**: Conceptualization; Resources; Supervision; Validation; Writing review & editing. **M. Shane Chapman**: Conceptualization; Resources; Supervision; Writing review & editing.

## ETHICS STATEMENT

Reviewed and approved by Dartmouth‐Hitchcock Health IRB; Approval #D19030.

## Supporting information

Supporting Information S1

## Data Availability

Dara are available on reasonable request from the authors.
