# Supplementary material for: Equivalent efficacy of indoor daylight and lamp‐based 5‐aminolevulinic acid photodynamic therapy for treatment of actinic keratosis
Source: Skin Health Dis. 2023 Mar 31;3(4):e226. doi: 10.1002/ski2.226 (PMC10395623; doi:10.1002/ski2.226)
Supplement: Supplementary file 1 — Supporting Information S1 [file SKI2-3-e226-s001.docx]

**SUPPLEMENTAL MATERIAL**

**Article Title**: Equivalent efficacy of indoor daylight and conventional ALA photodynamic therapy for treatment of actinic keratosis

Alberto J. Ruiz, PhD^1^, Ethan P.M. LaRochelle, PhD^1^, Marie-Christine P. Fahrner, RN^2^, Jennifer A. Emond,^3^ PhD, Kimberley S. Samkoe, PhD^1^, Brian W. Pogue, PhD^1^, M. Shane Chapman, MD^2^

^1^Thayer School of Engineering at Dartmouth, Hanover NH 03755

^2^Department of Dermatology, Geisel School of Medicine at Dartmouth, Lebanon NH 03755

^3^Dartmouth Geisel School of Medicine, Hanover NH 03755


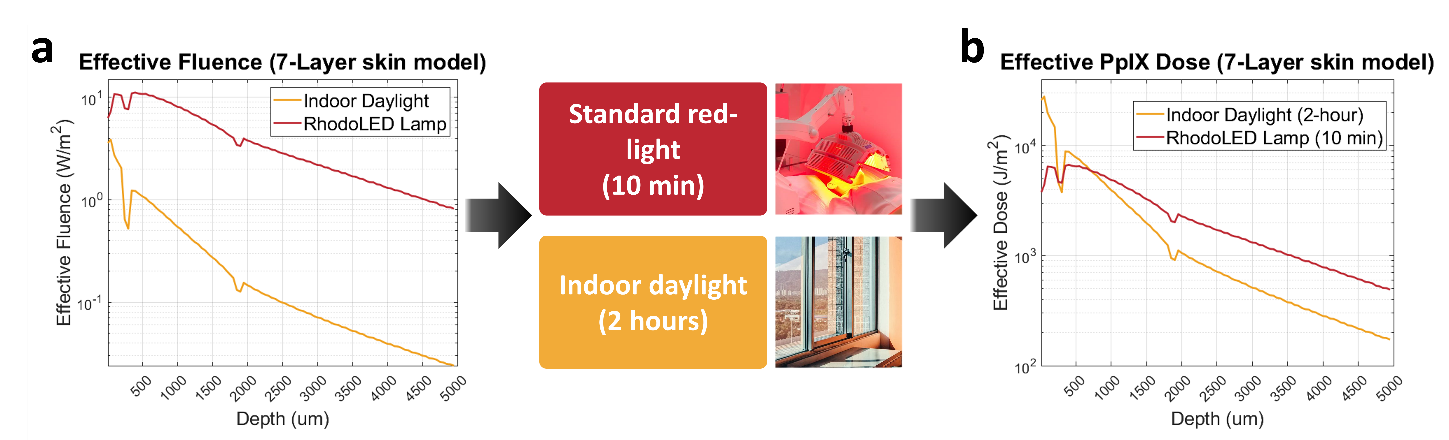


Supplemental Figure A1: Calculated fluence and dose curves for the red-lamp and indoor daylight PDT treatments. **(a)** Effective fluence and **(b)** effective PpIX dose for red-lamp (10 min) and indoor-daylight (2 hr) treatments.


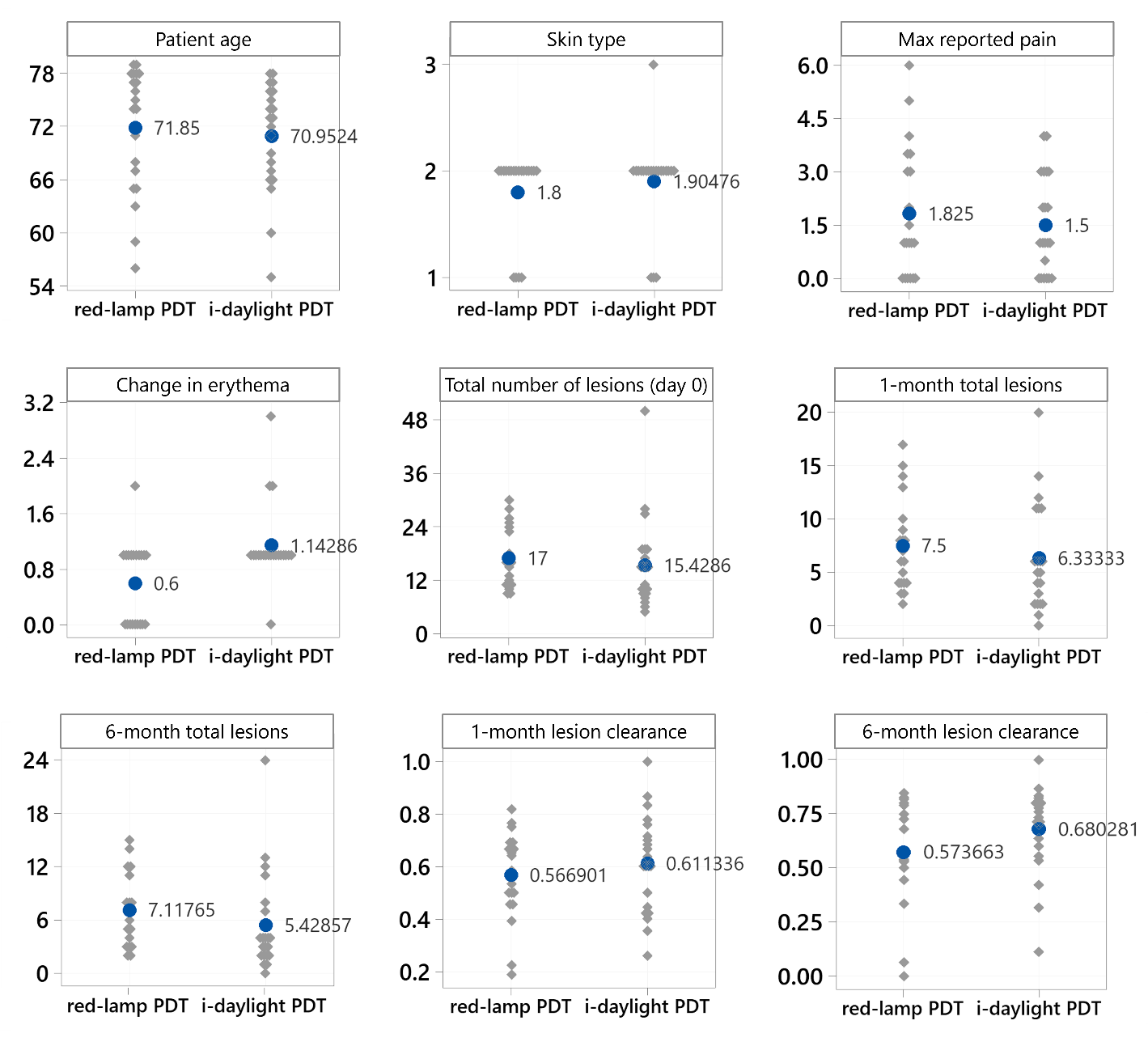


Supplemental Figure A2: Individual value plots for the patient characteristics and PDT treatment outcomes presented in Table II.


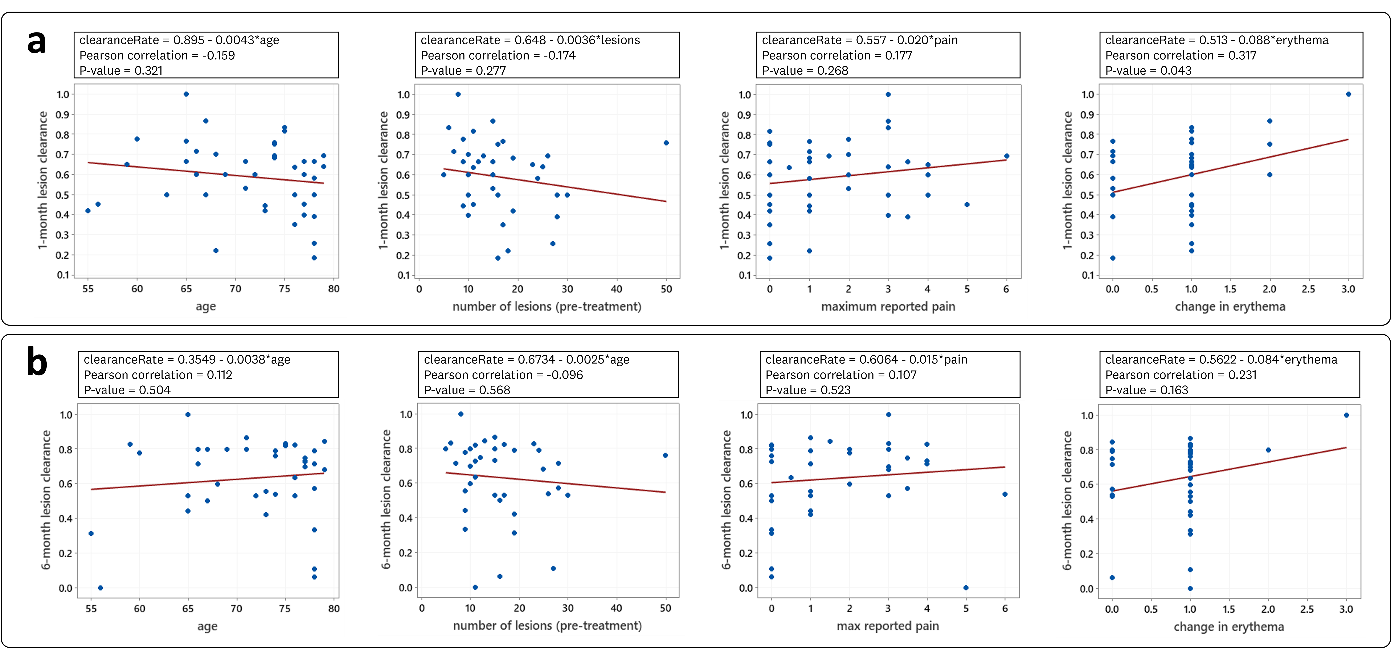


Supplemental Figure 4: Linear regression fits alongside corresponding Pearson correlation coefficient and P-values for the entire cohort (a) 1-month AK clearance rates and (b) 6-month AK clearance rate as related to age, number of pre-treatment lesions, max reported pain, and change in erythema.


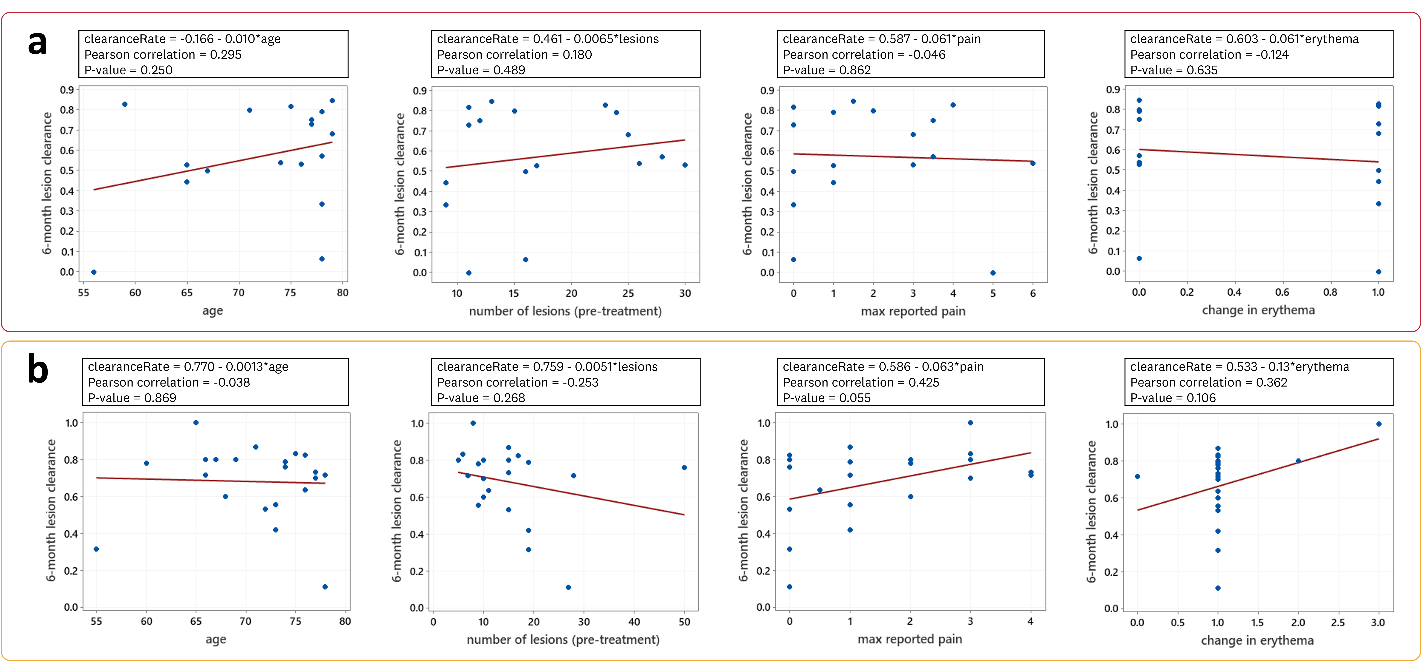


Supplemental Figure 5: Linear regression of each treatment cohort for 6-month AK lesion clearance rates as related to age, number of pre-treatment lesions, max reported pain, and change in erythema. (a) Red-lamp treatment showed had no statistically significant predictors with minimal to no correlation, and (b) the indoor-daylight treatment cohort showed statistical significance for the pain linear regression with a Pearson correlation coefficient = 0.425.


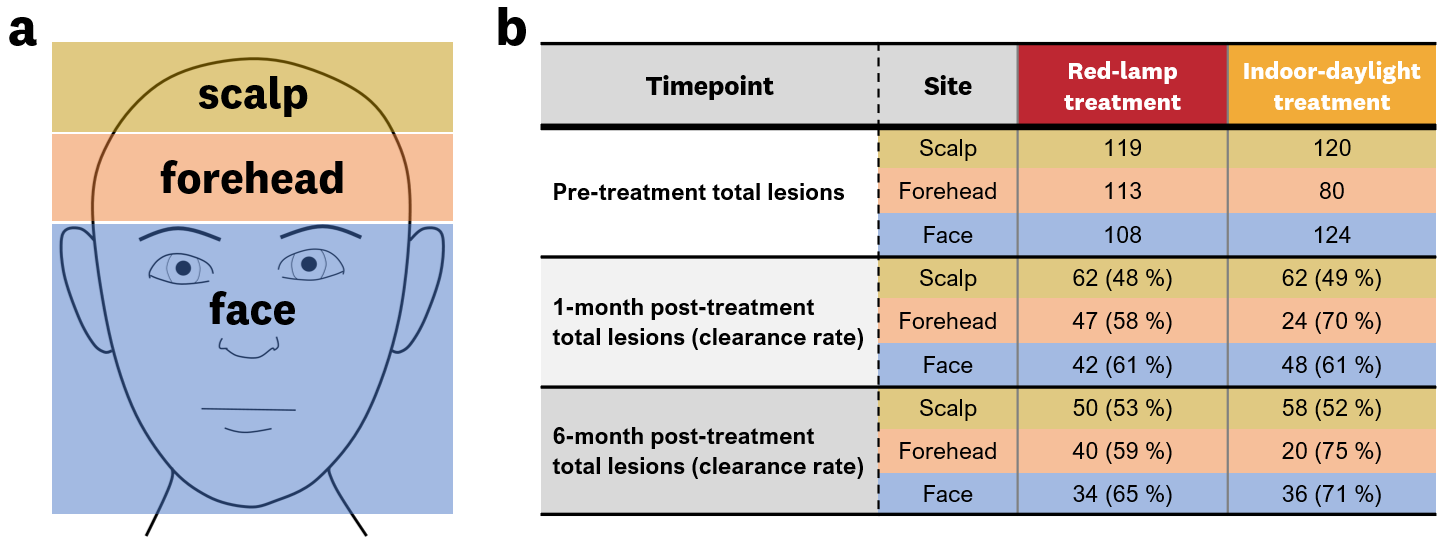


Supplemental Figure 6: Schematic depicting the location of lesions for classifying as scalp, forehead, and face.

**Supplemental Table I.** Site-specific lesion numbers and clearance for each treatment arm for 1 month and 6 month timepoints

| **Timepoint** | **Site** | **Red-lamp treatment** | **Indoor-daylight treatment** |
| --- | --- | --- | --- |
| **Pre-treatment total lesions** | Face | 108 | 124 |
|  | Forehead | 113 | 80 |
|  | Scalp | 119 | 120 |
| **1-month post-treatment total lesions (clearance rate)** | Face | 42 (61 %) | 48 (61 %) |
|  | Forehead | 47 (58 %) | 24 (70 %) |
|  | Scalp | 62 (48 %) | 62 (49 %) |
| **6-month post-treatment total lesions (clearance rate)** | Face | 34 (65 %) | 36 (71 %) |
|  | Forehead | 40 (59 %) | 20 (75 %) |
|  | Scalp | 50 (53 %) | 58 (52 %) |

**Study#19030 Patient #:__ __**

**Incubation Pain Rating**

| **0 min Incubation - Pain Rating** | | | | | | | | | | |
| --- | --- | --- | --- | --- | --- | --- | --- | --- | --- | --- |
| **0** | **1** | **2** | **3** | **4** | **5** | **6** | **7** | **8** | **9** | **10** |

| **30 min Incubation - Pain Rating** | | | | | | | | | | |
| --- | --- | --- | --- | --- | --- | --- | --- | --- | --- | --- |
| **0** | **1** | **2** | **3** | **4** | **5** | **6** | **7** | **8** | **9** | **10** |

**Dailight PDT Pain Rating**

| **30 min dPDT Pain Rating** | | | | | | | | | | |
| --- | --- | --- | --- | --- | --- | --- | --- | --- | --- | --- |
| **0** | **1** | **2** | **3** | **4** | **5** | **6** | **7** | **8** | **9** | **10** |

| **1hr dPDT Pain Rating** | | | | | | | | | | |
| --- | --- | --- | --- | --- | --- | --- | --- | --- | --- | --- |
| **0** | **1** | **2** | **3** | **4** | **5** | **6** | **7** | **8** | **9** | **10** |

| **1hr30 min dPDT Pain Rating** | | | | | | | | | | |
| --- | --- | --- | --- | --- | --- | --- | --- | --- | --- | --- |
| **0** | **1** | **2** | **3** | **4** | **5** | **6** | **7** | **8** | **9** | **10** |

| **2hr dPDT Pain Rating** | | | | | | | | | | |
| --- | --- | --- | --- | --- | --- | --- | --- | --- | --- | --- |
| **0** | **1** | **2** | **3** | **4** | **5** | **6** | **7** | **8** | **9** | **10** |

**Red PDT Pain Rating**

| **5 min PDT Pain Rating** | | | | | | | | | | |
| --- | --- | --- | --- | --- | --- | --- | --- | --- | --- | --- |
| **0** | **1** | **2** | **3** | **4** | **5** | **6** | **7** | **8** | **9** | **10** |

| **10 min PDT Pain Rating** | | | | | | | | | | |
| --- | --- | --- | --- | --- | --- | --- | --- | --- | --- | --- |
| **0** | **1** | **2** | **3** | **4** | **5** | **6** | **7** | **8** | **9** | **10** |

**Staff Name: ___________________ Staff Signature: ________________________ Date: ____________**


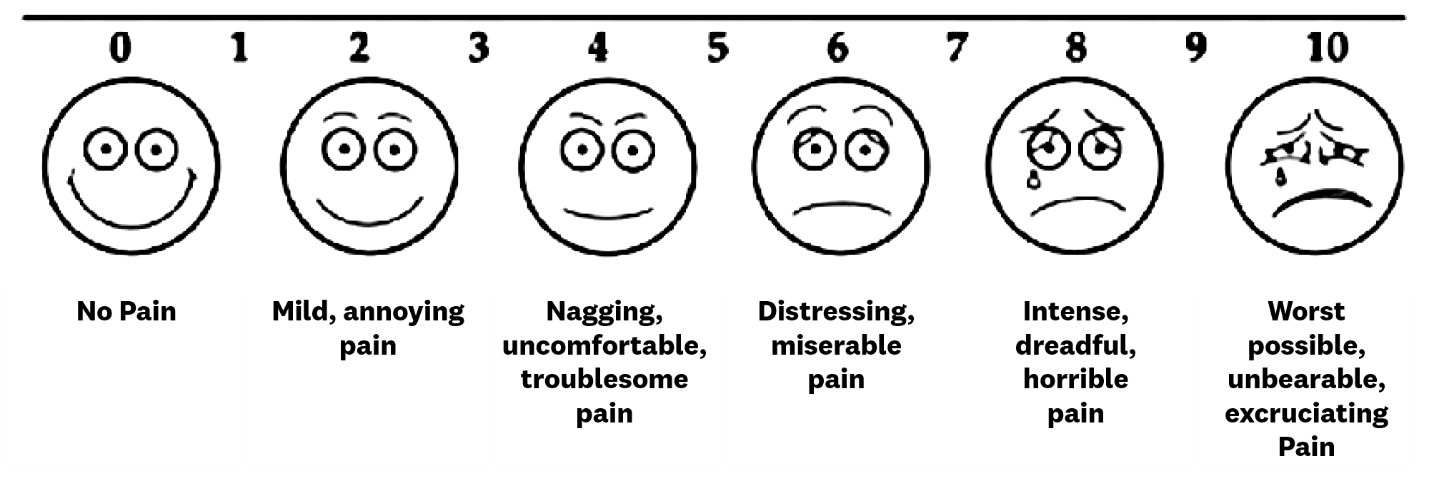


Protocol: **Indoor Daylight PDT for AK**

PI: M. Shane Chapman, MD Pt. Initials: _____________ Subject #: ______________

**PRE & POST-TREATMENT SAFETY ASSESSMENTS**

**(Circle one)**

**PRE-TREATMENT or POST-TREATMENT**

Visit #: ___________________________________________ Date: ____________________________

A study physician will complete post-treatment assessments based upon visual observation of swelling, erythema, and flaking/scaling. The assessment will be based upon the scaling system below:

| Swelling | Grade 0: not present  Grade 1: slight, lesion specific edema  Grade 2: palpable edema extending beyond individual lesions  Grade 3: confluent or visible edema  Grade 4: marked swelling, very easily noticeable from a distance |
| --- | --- |
| Erythema | Grade 0: not present  Grade 1: slightly pink <50%  Grade 2: pink or light red >50%  Grade 3: red 50-75%  Grade 4: severe redness, >75%, very easily noticeable from a distance |
| Flaking/Scaling | Grade 0: not present  Grade 1: isolated scale, specific to lesions  Grade 2: scale <50%  Grade 3: scale >50%, but <75%  Grade 4: scale >75%, very easily noticeable from a distance |

PI Signature: _____________________________________Date:_________________________________
